# Endometrial Dysbiosis: A Possible Association with Estrobolome Alteration

**DOI:** 10.3390/biom14101325

**Published:** 2024-10-18

**Authors:** Giorgia Scarfò, Simona Daniele, Elisa Chelucci, Francesca Papini, Francesco Epifani, Maria Ruggiero, Vito Cela, Ferdinando Franzoni, Paolo Giovanni Artini

**Affiliations:** 1 Division of General Medicine, Department of Clinical and Experimental Medicine, University of Pisa, 56126 Pisa, Italy; g.scarfo1@studenti.unipi.it (G.S.); ferdinando.franzoni@unipi.it (F.F.); 2 Department of Pharmacy, University of Pisa, 56126 Pisa, Italy; e.chelucci@studenti.unipi.it; 3 Division of Gynecology and Obstetrics, Azienda Ospedaliero Universitaria Pisana and Department of Clinical and Experimental Medicine, University of Pisa, 56126 Pisa, Italy; papinifra@libero.it (F.P.); celav2001@gmail.com (V.C.); 4 Department of Juridical and Economic Sciences, Pegaso Telematic University, 80143 Napoli, Italy; francesco.epifani@istitutofanfani.it; 5 Fanfani, Diagnostics and Health, 50129 Firenze, Italy; 6 San Rossore Clinic Care, 56100 Pisa, Italy; fertility@sanrossorecura.it

**Keywords:** endometrial dysbiosis, women infertility, estrobolome, inflammation

## Abstract

Background/Objectives: Microbiota modification at the endometrial level can favor gynecological diseases and impair women’s fertility. The overgrowth of pathogen microorganisms is related to the contemporary alteration of estrogen-metabolizing bacteria, including β-glucuronidase, thereby enhancing estrogen-related inflammatory states and decreasing anti-inflammatory cells. The possible connection between estrobolome impairment and gynecological diseases has been suggested in animal models. Nevertheless, in humans, coherent evidence on the estrobolome alteration and functionality of the female reproductive tract is still lacking. The objective of this study was to explore alterations in estrogen-related signaling and the putative link with endometrial dysbiosis. Methods: Women with infertility and repeated implantation failure (RIF, N = 40) were enrolled in order to explore the putative link between estrogen metabolism and endometrial dysbiosis. Endometrial biopsies were used to measure inflammatory and growth factor molecules. β-glucuronidase enzyme activity and estrogen receptor (ER) expression were also assessed. Results: Herein, increased levels of inflammatory molecules (i.e., IL-1β and HIF-1α) and decreased levels of the growth factor IGF-1 were found in the endometrial biopsies of patients presenting dysbiosis compared to eubiotic ones. β-glucuronidase activity and the expression of ERβ were significantly enhanced in patients in the dysbiosis group. Interestingly, Lactobacilli abundance was inversely related to β-glucuronidase activity and to ERβ expression, thus suggesting that an alteration of the estrogen-activating enzyme may affect the expression of ERs as well. Conclusions. Overall, these preliminary data suggested a link between endometrial dysbiosis and estrobolome impairment as possible synergistic contributing factors to women infertility and RIF.

## 1. Introduction

Human microbiota refers to the pool of microorganisms, including bacteria, viruses, and fungi, that physiologically colonize both our body’s surface and internal organs. These non-pathogen species play a crucial role in maintaining healthy conditions, such as effective immune activity and proper metabolic function [1,2].

A disbalance between commensal microorganisms and inflammatory ones, as well as an excessive overgrowth of certain species over others, alters microbiota homeostasis and leads to a state of dysbiosis. The disruption of gut microbiota is an unfavorable condition since it has been proven to negatively affect the immune response by causing intolerance to ingested proteins and to other nutrients and autoimmune disorders, including inflammatory bowel diseases and cancer. In addition, abnormal gut microbial diversity has been largely associated with metabolic syndromes and gynecological diseases, including endometriosis [3,4,5,6].

The systemic impact of the intestinal dysbiosis is primarily due to the alterations in the mucosal layer [5]; indeed, the disbalance of commensal microorganism abundance disrupts epithelial cells, and the consequent impairment of the intestinal barrier favors bacteria and endotoxin translocation [7]. This phenomenon establishes a pro-inflammatory state and alters the physiological sets of metabolites, facilitating new disease onset and the worsening of pre-existing conditions [1]. It has also to be considered that several intestinal bacteria have a key role in the transport of estrogen-metabolizing enzymes [8,9] and represent the estrobolome, intended as the pool of gut microorganisms involved in estrogen metabolism [10]. Particularly, gut bacteria species with β-glucuronidase (GUS) enzymes primarily affect estrogen metabolism due to their ability to deconjugate estrogens, thereby promoting their intestinal reabsorption and entry into the bloodstream [1]. The active deconjugated hormonal form can subsequently bind estrogen receptor alpha (ERα) and estrogen receptor beta (ERβ) and regulate different intracellular pathways, as well as the transcriptional activity of several genes related to estrogen signaling [11]. These signaling cascades finally affect various tissues, influencing reproductive organ health and their homeostasis [12,13]. Therefore, abnormalities in the gut microbiota composition impair β-glucuronidase enzyme activity, causing alterations in circulating estrogen levels and pathological conditions related to hyper- or hypo-estrogenism [5].

Similarly, microbiota modification or disruption at the endometrial level can favor gynecological diseases and impair women’s fertility: the overgrowth of several microorganisms, including Gardnerella, Ureoplasma, Enterococcus, Staphylococcus, E. coli, and the contemporary lack of estrogen-metabolizing bacteria, enhances estrogen-related inflammatory states and decreases anti-inflammatory cells (T-regs and uNKs), sensitive instead to progesterone activity [13,14].

Murine models have largely demonstrated the strict connection between estrobolome impairment and gynecological diseases: gut dysbiosis and alterations in fecal metabolites have been found in mice 42 days after endometriosis onset [12,15]. In addition, broad spectrum antibiotics have been shown to be useful in containing the worsening of endometriosic lesions and their subsequent inflammation [16]. However, dysbiosis can be observed only in advanced stages of the disease [12,13].

In humans, despite remarkable findings that led to the better characterization of endometriosis, little is still known about the correlation between gut or endometrial microbiota and alterations of the female reproductive tract [5,17,18].

Herein, a population of women with infertility and repeated implantation failure were enrolled in order to explore the putative link between estrogen metabolism and endometrial dysbiosis in infertile women.

## 2. Materials and Methods

### 2.1. Patients

Patients (N = 40) with an age range comprising between 35 and 41 years and subjected to IVF treatment at the Centre for Infertility and Assisted Reproduction of the Department of Clinical and Experimental Medicine of Pisa were recruited. This sample also included the 26 women who had been enrolled in our previous study [19]. After an accurate clinical history and physical examination, biochemical analyses and transvaginal ultrasonography were performed. The Guidelines of the Declaration of Helsinki were applied, and the study was approved by the Ethics Committee (CTO, Clinical Trial Office) of Azienda Ospedaliero Universitaria Pisana (AOUP) (protocol code 35105, approved on 13 June 2019) [19].

IVF treatment and biopsies sampling were conducted between June 2019 and April 2022. Enrolled women suffered from primary infertility (unexplained primary infertility (N = 12), tubal factor (N = 28) for at least 3 years and were characterized by three or more failed embryo transfers with good-quality embryos (i.e., presenting RIF, repeated implantation failure).

Women with obesity who smoked and had secondary infertility were excluded from the study, as well as those with evidence of uterine diseases (endometriosis, polyps, fibroids, cancer), autoimmune or endocrinological disorders (including thyroiditis or polycystic ovarian syndrome), or presenting fallopian tube effusion and pelvic inflammatory diseases. Other exclusion criteria were varicocele or azoospermia of the male partner and couples undergoing ICSI.

### 2.2. Sample Collection

In order to detect the number of Lactobacilli, an endometrial biopsy was performed after endometrial preparation to achieve an adequate endometrium thickness. Endometrial biopsy samples were collected on natural cycles (18–22 days) or on artificial hormone cycles (progesterone + 5 days) [19].

A MedGyn IV pipette (MedGyn, Addison, IL, USA) was used to sample the tissue, preventing the contamination and degradation of the genetic material, according to endometrial microbiome genomic analysis instructions (for details, see [19]. Samples were later stored at −80 °C until processing for DNA extraction and protein analyses.

Women were categorized into two groups based on *Lactobacilli* percentage in the endometrium: the eubiosis group referred to patients with a percentage of lactobacilli ≥ 90%; values < 90% defined a condition of endometrial dysbiosis [2]. Once divided, further analyses were performed and reported [19].

### 2.3. Bacterial DNA Extraction and RT-PCR

Endometrial tissues were used to detect the total bacterial DNA using a QIAamp^®^ DNA Microbiome Kit (QIAGEN, Hilden, Germany) following the manufacturer’s protocol [19] and further quantified using NanoDrop (Thermofisher, Waltham, MA, USA). The amplification of the DNA was made using a MiniOpticon (BIORAD, Milano, Italy). RT-PCR reactions consisted of 10 μL of Fluocycle^®^ II SYBR^®^ (Euroclone, Milan, Italy), 0.6 μL of 10 μM forward and reverse primers, 5 μL of cDNA, and 3.8 μL of H2O. All reactions were conducted for 40 cycles using the following temperature profiles: 98 °C for 30 s (initial denaturation); T °C for 30 s (annealing); and 72 °C for 3 s (extension).

### 2.4. β-Glucoronidase Activity Assay

A fluorometric commercial kit (ab234625, Abcam, Prodotti Gianni, Milan, Italy) was used to evaluate β-Glucoronidase enzyme activity in the endometrial tissue samples, following the manufacturer’s instructions. Briefly, tissue samples were homogenized mechanically (sonification, 35A), after adding 100 µL β-Glucoronidase Assay Buffer to 10 mg (wet weight) of each biopsy. Subsequently, tissue lysates were centrifuged at 10000× *g* for 5 min at 4 °C, and the supernatants were detected to be assayed. For each reaction, 10 µL of samples were added into a black 96-well plate, adjusting the volume to 90 µL with Assay Buffer. Finally, fluorescence intensity was measured (Ex/Em = 330/450 nm) immediately after the addition of the substrate (10 µL) for 0–60 min (kinetic mode) at 37 °C. The provided substrate is specific to β-Glucoronidases, and it is cleaved into a fluorescent product in the enzyme’s presence. A standard curve (4-Methylumbelliferone, 4-MU) was used to calculate the 4-MU amount (pmol) in samples, generated during the reaction time (Δt = 60 min − 0 min). The quantification of samples’ β-Glucoronidase activity (pmol/min/mL= µU/mL) was obtained by applying the following formula: β-Glucoronidase activity= (B/Δt × V) × D, where B is the amount of 4-MU in the samples (pmol); Δt is the reaction time (min); V refers to the sample volume (mL), and D is the sample dilution factor. Our samples were not diluted before the reaction set up. The results were expressed as µU/mL, where 1 unit is the amount of enzyme able to cleave 1 µmol of substrate/min at 37 °C.

### 2.5. Interleukines (ILs) and Signaling Molecules in Endometrial Biopsies

Enzyme-linked immunosorbent assay (ELISA) kits (SEA079Hu for IL-6, SEA080Hu for IL-8, SEA111Hu for IL-12, and SEA056Hu for IL-10) were used to analyze the levels of interleukins, according to the manufacturer’s instructions. Specifically, 100 µL of endometrial tissue homogenate containing 10 µg of total proteins was put into the appropriate wells and incubated for 1 h at 37 °C. After the incubation time, 100 µL of primary antibody was added for 1 h at 37 °C. The solution was later washed; 100 µL of secondary antibody was incubated for 30 min at 37 °C and then added to each well, leaving the color to develop for 10–20 min at 37 °C. Absorbance was measured at 450 nm.

Similarly, HIF-1α (RAB1057-1KT, SigmaAldrich, Milan, Italy), COX-2 (ab267646, Prodotti Gianni, Milan, Italy), and IGF-1 (ab108873, Prodotti Gianni, Milan, Italy) were analyzed using an ELISA kit following the manufacturer’s instructions. The data were presented as pg/µg total proteins present in the endometrial sample.

### 2.6. Expression of Estrogen Receptors

Aliquots of the biopsies were lysed and used for the measurement of ERα and ERβ in the obtained tissue homogenates using a specific competitive ELISA kit, following the manufacturer’s instructions (ab277408 and ab285292, Prodotti Gianni, Milan, Italy).

### 2.7. Statistical Analysis

To perform data analysis and graphical representations, GraphPad Prism 8 (GraphPad Software Inc., San Diego, CA, USA) was used. For data analysis and graphical presentations, all data were reported as mean ± SEM. The unpaired *t*-test was used for statistical analysis, while linear regression analysis was used to correlate variables. All statistical procedures were performed using the StatView program (Abacus Concepts, Inc., SAS Institute, Cary, NC, USA) [20].

## 3. Results

### 3.1. Descriptive Statistics

The clinical features of recruited patients are shown in Table 1. There were no significant differences in age and BMI between the two categories. The number of years of infertility, trials, and good-quality embryos, and the rate of good-quality embryos over the number of trials were comparable (Table 1).

As expected, dysbiotic patients had significantly lower lactobacillus species in the endometrial biopsies compared to eubiotic ones (*p* < 0.0001).

Next, we investigated the expression levels of selected key inflammatory (IL-1 β, IL-6, IL-8, HIF-1α, cox-2), anti-inflammatory (i.e., IL-10), and molecules or growth factors (IGF-1) in the endometrial species, as reported in Table 2.

Considering the inflammatory molecules, dysbiotic patients had significantly increased levels of IL-1 β (*p* < 0.0001) and HIF-1α (*p* = 0.0053) compared to eubiotic ones. In contrast, the eubiosis group showed higher levels of IGF-1 (*p* = 0.0279). No significant differences were found in the IL-6 (*p* = 0.5662), IL-8 (*p* = 0.3104), and IL-10 (*p* = 0.1758) levels between the two groups. Similarly, COX-2 expression was found to be comparable (*p* = 0.9949).

To investigate the involvement of the estrobolome system, we next investigated the activity of the β-glucuronidase enzyme and the expression of the ERs. Interestingly, the β-glucuronidase enzyme showed significantly higher activity in the dysbiosis group (*p* < 0.0001). Moreover, the expression of ERβ was significantly enhanced (*p* = 0.0044) in patients with dysbiosis with respect to patients with eubiosis. Conversely, no significant changes were evidenced for ERα (*p* = 0.3514) (Table 2).

### 3.2. Correlations Between Clinical and Biochemical Parameters

**Figure 1:**
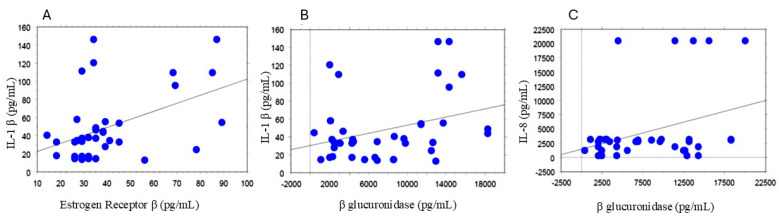
Correlations between ER β and IL−1 β (**A**); Correlations Between β glucuronidase and IL−1 β and IL−8 (**B**,**C**). All correlations between the selected variables were performed using simple linear regression analysis using the StatView program (Abacus Concepts, Inc., SAS Institute, Cary, NC, USA). *p* and R2 values obtained for each correlation are reported in the text.

All clinical and biochemical parameters were correlated using a simple regression analysis. Interestingly, IL-1β quantity correlated directly with the expression of ERβ (*p* = 0.0035; R2 = 0.208, Figure 1A), as well as with β-glucuronidase activity (*p* = 0.0460; R2 = 0.103, Figure 1B). Similarly, the endometrial levels of IL-8 positively correlated with those of β-glucuronidase (*p* = 0.0387; R2 = 0.11, Figure 1C).

**Figure 2:**
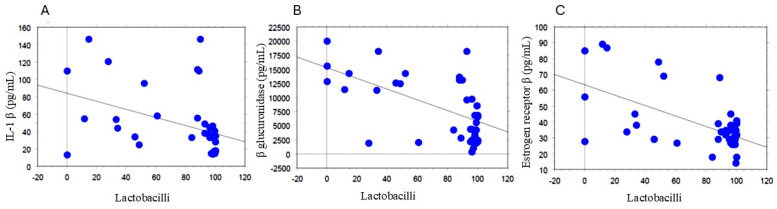
Correlations of Lactobacilli and IL−1 β (**A**), β glucuronidase (**B**), and ER β (**C**). All correlations between the selected variables were performed by simple linear regression analysis using the StatView program (Abacus Concepts, Inc., SAS Institute, Cary, NC, USA). *p* and R2 values obtained for each correlation are reported in the respective panel.

Conversely, IL−1β negatively correlated with Lactobacilli abundance (*p* = 0.0104; R2 = 0.164, Figure 2A). Accordingly, Lactobacilli abundance was inversely related to β- glucuronidase activity (*p* < 0.0001; R2 = 0.351, Figure 2B), and ER-β (*p* < 0.0001; R2 = 0.346, Figure 2C).

## 4. Discussion

In the present study, we investigated if the alteration of the endometrial microbiota could be related to a different pattern in the estrogen metabolism and receptor expression in women with repeated implantation failure. The main findings of the study were as follows: (i) increased levels of inflammatory molecules (i.e., IL-1β and HIF-1α) and decreased levels of the growth factor IGF-1 were found in the endometrial biopsies of patients presenting endometrial dysbiosis compared to eubiotic ones; (ii) patients with endometrial eubiosis presented significantly higher levels of IGF-1; (iii) β glucuronidase activity and the expression of ERβ were significantly enhanced in patients in the dysbiosis group. Interestingly, Lactobacilli abundance was significantly inversely related to β-glucuronidase activity and to ERβ expression, thus suggesting that an alteration of the estrogen-activating enzyme may affect the expression of ERs as well. Overall, these preliminary data suggested a link between endometrial dysbiosis and estrobolome impairment as possible synergistic contributing factors to women’s infertility and repeated implantation failure.

Microbiota modification or disruption at the endometrial level can favor gynecological diseases and impair women’s fertility. The overgrowth of pathogen microorganisms is related to the contemporary alteration of estrogen-metabolizing bacteria, thereby enhancing estrogen-related inflammatory states and decreasing anti-inflammatory cells [1,14]. Therefore, abnormalities in the gut microbiota composition impair β-glucuronidase enzyme activity, causing alterations in circulating estrogen levels and pathological conditions related to hyper- or hypo-estrogenism [5]. In this sense, the strict connection between estrobolome impairment and gynecological diseases has been suggested by animal models, such as concern endometriosis, endometrial cancer, and so on [21,22]. Nevertheless, in humans, coherent evidence of estrobolome alteration and functionality of the female reproductive tract is still lacking.

Herein, a population of women with infertility and repeated implantation failure were enrolled in order to explore the putative link between estrogen metabolism and endometrial dysbiosis. The pathophysiology of RIF can be attributed to either embryonic factors, such as genetic or chromosomal abnormalities, maternal factors, or a disturbed endometrial-embryo crosstalk [23,24].

The enrolled population of infertile women was divided into two groups (i.e., eubiotic vs. dysbiotic patients) considering the microbiological composition revealed in the endometrial specimen. Accordingly, recent studies have shown that endometrial samples of women with RIF were non-*Lactobacillus* dominant, with an increased presence of *Streptococcus*, *Staphylococcus*, *Neisseria*, and *Klebsiella* [23,25]. These pathogens are known to compromise the integrity of the endometrial epithelial, which can lead to failed implantation [23,26]. Overall, several studies have hypothesized that a *Lactobacillus*-dominant microenvironment in the endometrium, if it exists at all, indicates an optimal environment for the embryo to implant [23,27].

During the adhesion process of the embryo to the endometrial lining, inflammatory mediators are carefully regulated [23,28]. In particular, the immune system is activated via pattern recognition receptors present on the endometrial cells; for this reason, the presence of pathogenic bacteria may lead to the dysregulation of cytokine levels in immune cell activation and negatively affect the local immune environment [23,29], finally impairing embryo implantation [23,30]. For this reason, we decided to measure the expression levels of selected key inflammatory (IL-1 β, IL-6, IL-8, HIF-1α, COX-2), anti-inflammatory (i.e., IL-10), molecules, or growth factors (IGF-1) in the endometrial species.

Herein, patients presenting endometrial dysbiosis presented significant elevated levels of IL-1 β compared to patients with endometrial eubiosis, and IL-1β was inversely correlated with Lactobacilli abundance, thus suggesting a strict link between the cytokine expression and the insurgence of endometrial dysbiosis. In this sense, a recent paper has shown that IL-1β plays a pivotal role in endometrial cell cycle/viability, and represents, along with the JNK signaling pathway, a pathogenetic target to mitigate the potential to enhance uterine receptivity [31].

Moreover, dysbiotic patients displayed significantly elevated expressions of HIF-1α. In accordance, levels of HIF-1α in endometrial biopsies have shown a significant decrease in RIF patients when compared to the control group not presenting RIF [32], overall confirming the importance of HIF expression in reducing endometrial receptivity.

The eubiosis group showed significantly higher levels of IGF-1, as reported previously [19]. In accordance, patients with RIF have revealed decreased levels of IGF-1 binding protein [32], and experiments in mice have revealed the stimulating effects of IGF-1 on preimplantation embryos after their cryopreservation [33], further confirming the positive role of this growth factor in mediating the crosstalk between embryos and endometrium.

Finally, no significant differences were found between the IL-6, IL-8, COX-2, and IL-10 levels between the two groups. Of note, our previous study evidenced that women with dysbiosis exhibited a significant enhancement of inflammatory markers (IL-6 and COX-2), as well as a decrease in anti-inflammatory factors, IL-10, with respect to the eubiosis group [19]. These differences could be primarily ascribed to a probably different set of pathogens compared with those in the previous study. Unfortunately, in this paper, the endometrial presence of pathogens was not considered, and the sample was not large enough for adequate analysis to assess the presence of specific microbial species.

To investigate the involvement of the estrobolome system, we next investigated the activity of the β-glucuronidase enzyme and ER expression. Indeed, β-glucuronidase enzymes can deconjugate estrogens, thereby promoting their reabsorption and entry into the bloodstream [1]. Abnormalities in the gut microbiota composition can affect β-glucuronidase enzyme activity, causing alterations in circulating estrogen levels and pathological conditions related to hyper- or hypo-estrogenism [5]. Nevertheless, the role of the β-glucuronidase enzyme in the endometrial tissue and its relationship with gynecological diseases has been partially elucidated, in particular in humans.

Herein, the β-glucuronidase enzyme showed significantly higher activity in the endometrial samples of patients presenting dysbiosis. Interestingly, β glucuronidase activity was inversely related to Lactobacilli abundance. To the best of our knowledge, the current literature is lacking data on enzyme activity in infertile women. Nevertheless, microbial β-glucuronidase activity has been reported to be elevated in women with polycystic ovary syndrome [34]. Moreover, the gut microbiotas of endometriosis patients have been hypothesized to present a larger number of β-glucuronidase-producing bacteria [1]. Overall, the available data suggest the implication and hyperactivity of this enzyme in gynecological diseases.

The enhanced β-glucuronidase activity can be reasonably linked to an altered estrogen metabolism and signaling [35]. The active deconjugated hormonal form can subsequently bind ERα and ERβ, thereby affecting different signaling cascades that influence reproductive organ health and their homeostasis [12,13].

In our study, no significant changes were evidenced for ERα. In contrast, the expression of Erβ was significantly enhanced in patients with dysbiosis with respect to patients with eubiosis, and Lactobacilli abundance was inversely related to ER β.

High levels of ERβ can disbalance the ERβ/ERα ratio, which has been associated with inflammation in the stromal cells of patients with endometriosis and to promote endometriosis progression [36,37]. Interestingly, the expression of ERβ was directly related with β glucuronidase activity, thus suggesting that an alteration of estrogen-activating enzyme may affect the expression of ERs as well.

Although the study has emphasized the link between endometrial dysbiosis and estrobolome activity, some limitations should be underlined: first of all, the low number of patients was not sufficient for categorizing women based on the pathogen located in the endometrium. In addition, it was not possible to compare enrolled subjects with patients not presenting RIF because the latter do not need to undergo endometrial biopsy with microbiota analysis according to clinical procedures. Consequently, a comparison between the microbiota composition was not possible, nor was an analysis on the endometrial inflammatory status in women with and without RIF.

## 5. Conclusions

Our data showed that, among women presenting RIF, those who had significantly lower lactobacillus species in the endometrial biopsies also presented increased levels of inflammatory cytokines. Moreover, the dysbiotic group was characterized by the enhanced activity of both the β-glucuronidase enzyme and ERβ expression, thus suggesting an impaired estrogen metabolism. Interestingly, Lactobacilli abundance was significantly inversely related to β-glucuronidase activity and to ERβ expression, thus suggesting that an alteration of estrogen-activating enzyme may affect the expression of ERs as well. Compared with our previous study, in which the composition of the microbiota and its correlation with the levels of inflammatory cytokines were analyzed [19], the novelty of the present study lies in emphasizing the relationship between endometrial dysbiosis and the estrogen metabolism among women presenting RIF. Overall, our results highlight the strict link between alterations of endometrial microbiota and the estrobolome, laying the foundation for further studies on the use of probiotics and their possible effect in reestablishing both a physiological uterine microorganism pattern and an appropriate estrogen activity, fundamental to ensuring effective embryo implantation in patients with RIF.

## Figures and Tables

**Table 1:** Descriptive statistics of clinical parameters measured in eubiosis and dysbiosis women. Data are expressed as mean ± SD. Statistical analysis was performed using an unpaired *t*-test.

Parameters	Eubiosis (Mean ± SD)	Dysbiosis (Mean ± SD)
Age	38.1 ± 3.5	38.3 ± 2.9
BMI	21 ± 2.3	20 ± 2.7
Number of trials	2.4 ± 1.4	2.5 ± 1.4
Good-quality embryos	3.5 ± 2.7	2.6 ± 1.6
Years of infertility	4.3 ± 1.2	3.9 ± 1.8
Good-quality embryos/trials	1.5 ± 0.7	1.4 ± 1

**Table 2:** Biochemical parameters measured in the two study groups. Lactobacillus presence is expressed as the percentage of the microbial species versus the total; IL-1 β, IL-6, IL-8, IL 10, ERα, and ERβ are expressed as pg/mL; HIF-1α, IGF-1, and COX-2 are expressed as ng/µg; β glucuronidase is expressed as µU/mL. All data are expressed as mean ± standard deviation (mean ± SD). Statistical analysis was performed using an unpaired *t*-test. * *p* < 0.05, ** *p* < 0.01, *** *p* < 0.001, **** *p* < 0.0001 vs. eubiosis patients.

Parameters	Eubiosis (Mean ± SD)	Dysbiosis (Mean ± SD)
Lactobacillus (percentage)	98 ± 1.8	49.9 ± 34.3 ****
IL-1 β (pg/mL)	27.5 ± 11.4	70.4 ± 42.2 ****
IL-6 (pg/mL)	20.8 ± 20.6	16.2 ± 22.3
IL-8 (pg/mL)	3501.4 ± 4226	5574.3 ± 7754.8
IL-10 (pg/mL)	63.3 ± 20.2	47.5 ± 46.9
HIF-1α (ng/µg)	11.9 ± 10.1	22.9 ± 13.3 *
IGF-1 (ng/µg)	42.5 ± 41.9	18.2 ± 19.9 *
COX-2 (ng/µg)	51.2 ± 65.5	51.4 ± 67.7
β glucuronidase (µU/mL)	4675.3 ± 2820.9	11,726.7 ± 5224.6 ***
ERα (pg/mL)	127.9 ± 54.3	141.5 ± 34.8
ERβ (pg/mL)	31 ± 7.4	47.5 ± 23.2 **

## Data Availability

The data presented in this study are available on request from the corresponding author.
